# P02.176. Acupuncture for symptom management in hemodialysis patients: a prospective, observational pilot study

**DOI:** 10.1186/1472-6882-12-S1-P232

**Published:** 2012-06-12

**Authors:** K Kim, T Kim, J Kang, M Lee, J Kim, M Shin, S Jung, A Kim, K Kang, S Choi, G Yang, S Noh

**Affiliations:** 1 Korea Institute of Oriental Medicine, Daejeon, Republic of Korea; 2 Kyung Hee University, Seoul, Republic of Korea; 3 School of Korean Medicine, Pusan National University, Yangsan, Republic of Korea

## Purpose

Patients undergoing hemodialysis suffer from a variety of complications related to end-stage renal disease. This prospective, observational pilot study aims to determine the feasibility, safety, and possible benefits of acupuncture for symptom management in patients undergoing hemodialysis.

## Methods

Twenty-four patients undergoing hemodialysis received acupuncture treatment for their symptoms. Manually stimulated, individualized acupuncture treatments were provided twice a week for 6 consecutive weeks on a nondialysis day or on the day of hemodialysis prior to initiating treatment. Symptoms were evaluated using the Measure Your Medical Outcome Profiles 2 Questionnaire, and quality of life was measured by the Kidney Disease Quality of Life-Short Form (KDQOL-SF™) Version 1.3 at baseline, 7 weeks and 11 weeks from baseline. Statistical analysis was conducted on the basis of the intention-to-treat principle.

## Results

Twenty-one patients (87%) completed the whole treatment course and follow-up evaluation. Three patients dropped out due to increased fatigue (n=1), pancreatic and renal transplantation (n=1), and infections of the ateriovenous fistula used for hemodialysis access (n=1). Patients experienced a significant improvement of symptoms considered the most bothersome, reporting a decrease of 1.87 and 2.08 points on a 0-6 symptom scale at 7 weeks and 11 weeks, respectively (both p<0.0001). Some subscales of KDQOL-SF(™) showed significant improvement at 7 weeks (effects of kidney disease, burden of kidney disease, role-limitations physical, emotional well-being, and energy/fatigue) and 11 weeks (physical functioning and energy/fatigue). No serious adverse events related to acupuncture occurred.

## Conclusion

Acupuncture seems feasible and safe for symptom management in patients undergoing hemodialysis. Future controlled trials are needed to confirm the benefits of acupuncture.

